# Supplementary material for: Enantioselectivity of discretized helical supramolecule consisting of achiral cobalt phthalocyanines via chiral-induced spin selectivity effect
Source: Nat Commun. 2023 Jul 28;14:4530. doi: 10.1038/s41467-023-40133-z (PMC10382588; doi:10.1038/s41467-023-40133-z)
Supplement: Supplementary file 1 — Supplementary information [file 41467_2023_40133_MOESM1_ESM.pdf]

Supplementary Information

Enantioselectivity of discretized helical supramolecule consisting of achiral cobalt phthalocyanines via chiral-induced spin selectivity effect

Hiroki Aizawa[1,2], Takuro Sato[1,2*], Saori Maki-Yonekura[3], Koji Yonekura[3,4,5],

Kiyofumi Takaba[3], Tasuku Hamaguchi[3, #], Taketoshi Minato[1], and Hiroshi M. Yamamoto[1,2*]

[1]*Institute for Molecular Science, Myodaiji, Okazaki, 444-8585, Japan.*

[2]*the Graduate University for Advanced Studies, Myodaiji, Okazaki, 444-8585, Japan.*

[3]*Biostructural Mechanism Laboratory, RIKEN SPring-8 Center, Hyogo 679-5148, Japan.*

[4]*Institute of Multidisciplinary Research for Advanced Materials, Tohoku University, 2-1-1 Katahira, Aoba-ku, Sendai 980-8577, Japan*

[5]*Advanced Electron Microscope Development Unit, RIKEN-JEOL Collaboration Center, RIKEN Baton Zone Program, 1-1-1 Kouto, Sayo, Hyogo 679-5148, Japan*

# The present address is 4.

[*] To whom correspondence should be addressed.

Email: takurosato@ims.ac.jp, yhiroshi@ims.ac.jp

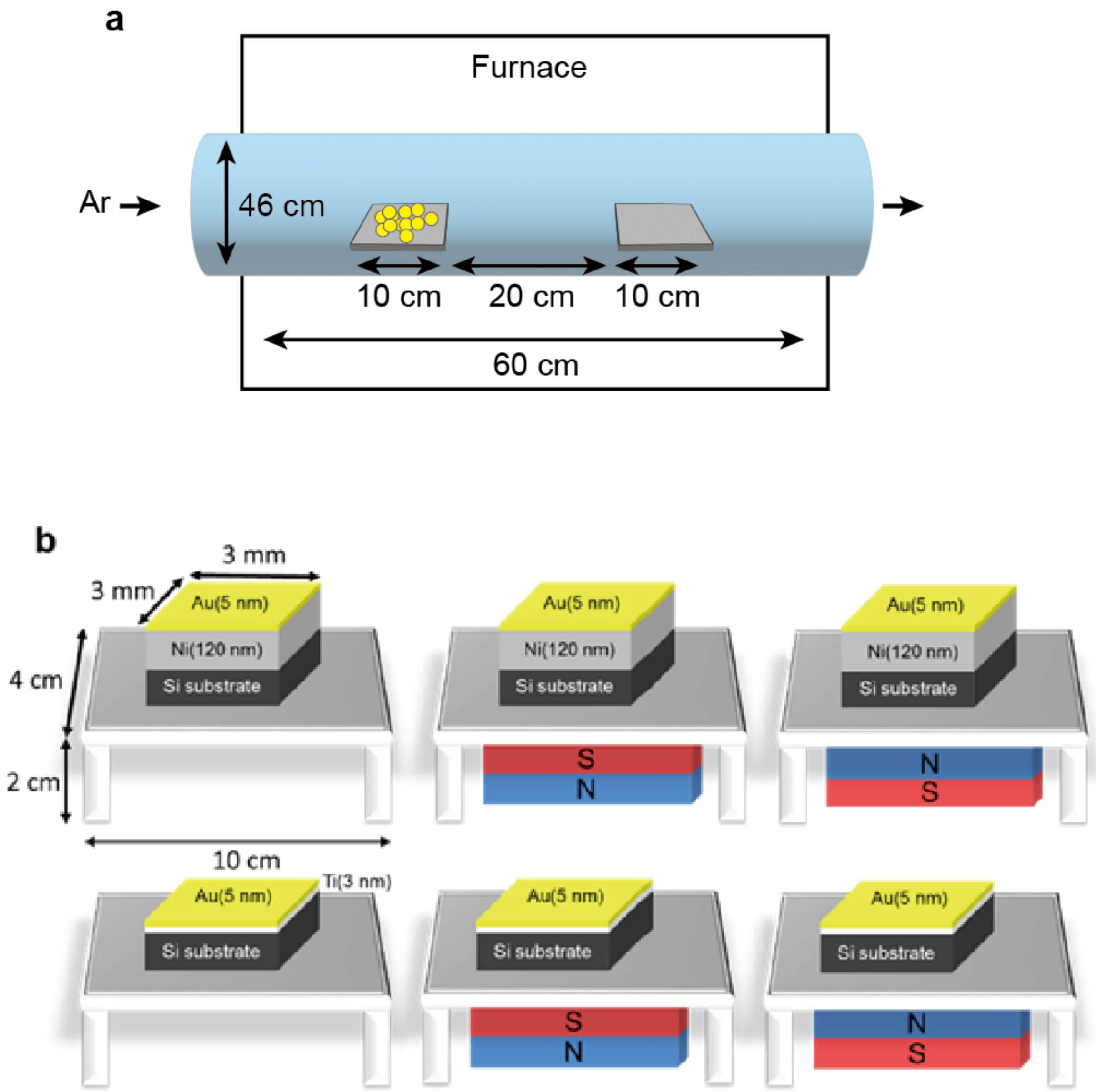

Supplementary Figure 1 │ Detailed configurations of our PVD setup and the substrates. a. Schematic image of PVD system with information of length scale. **b.** Geometry of the substrates and SmCo magnet placed at downstream side of the tube. The substrates covered with ferromagnetic or nonmagnetic metal and SmCo magnets are placed on top and bottom of the quartz boat, respectively.

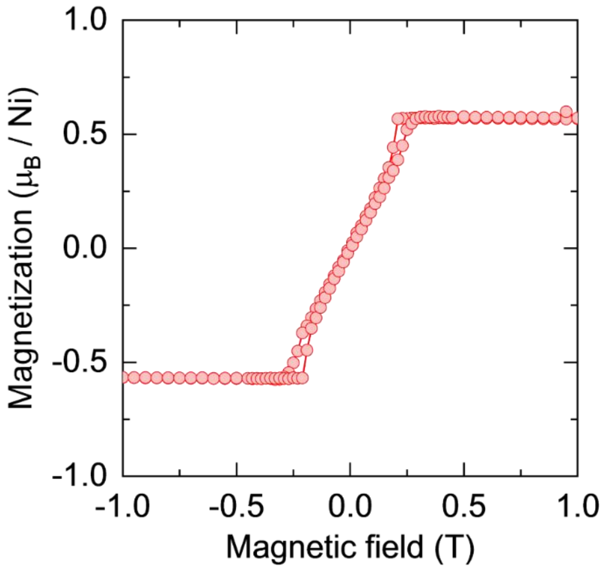

Supplementary Figure 2 │ *M-H* profile of the Ni film (120 nm) prepared on Si substrate.

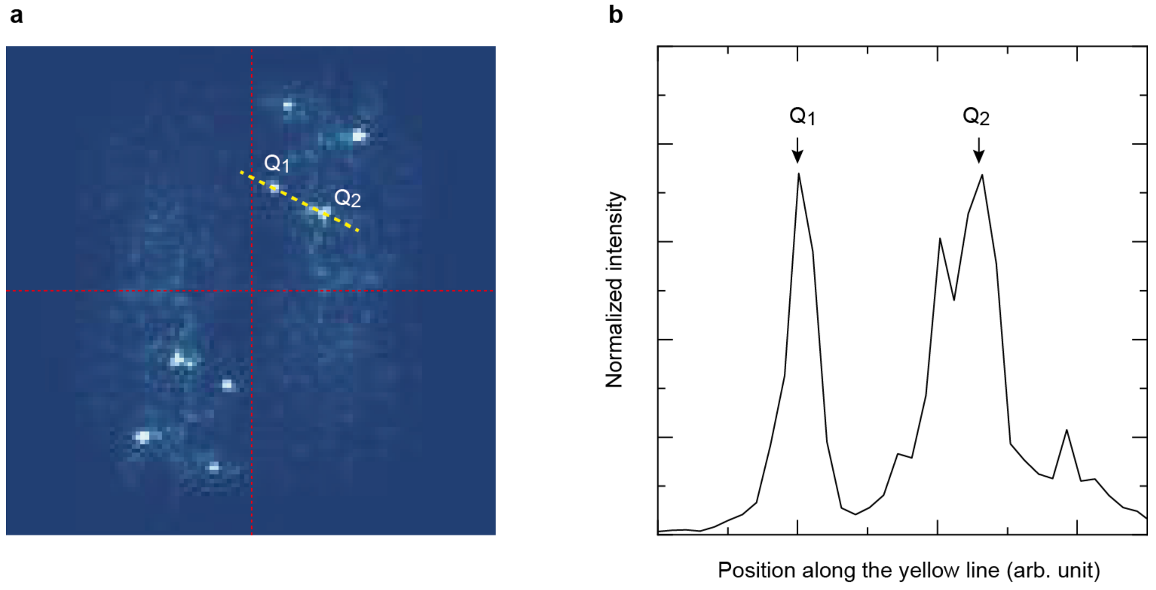

Supplementary Figure 3 │ Line profile in the FFT of an ET reconstruction along the Q_1 and Q_2 spots. a. A typical FFT pattern of the area including helical dislocation. It is reproduced from Fig. 3b in the main text. **b.** Line profile along the yellow dashed line in (a). Two well-separated peaks are clearly obtained.

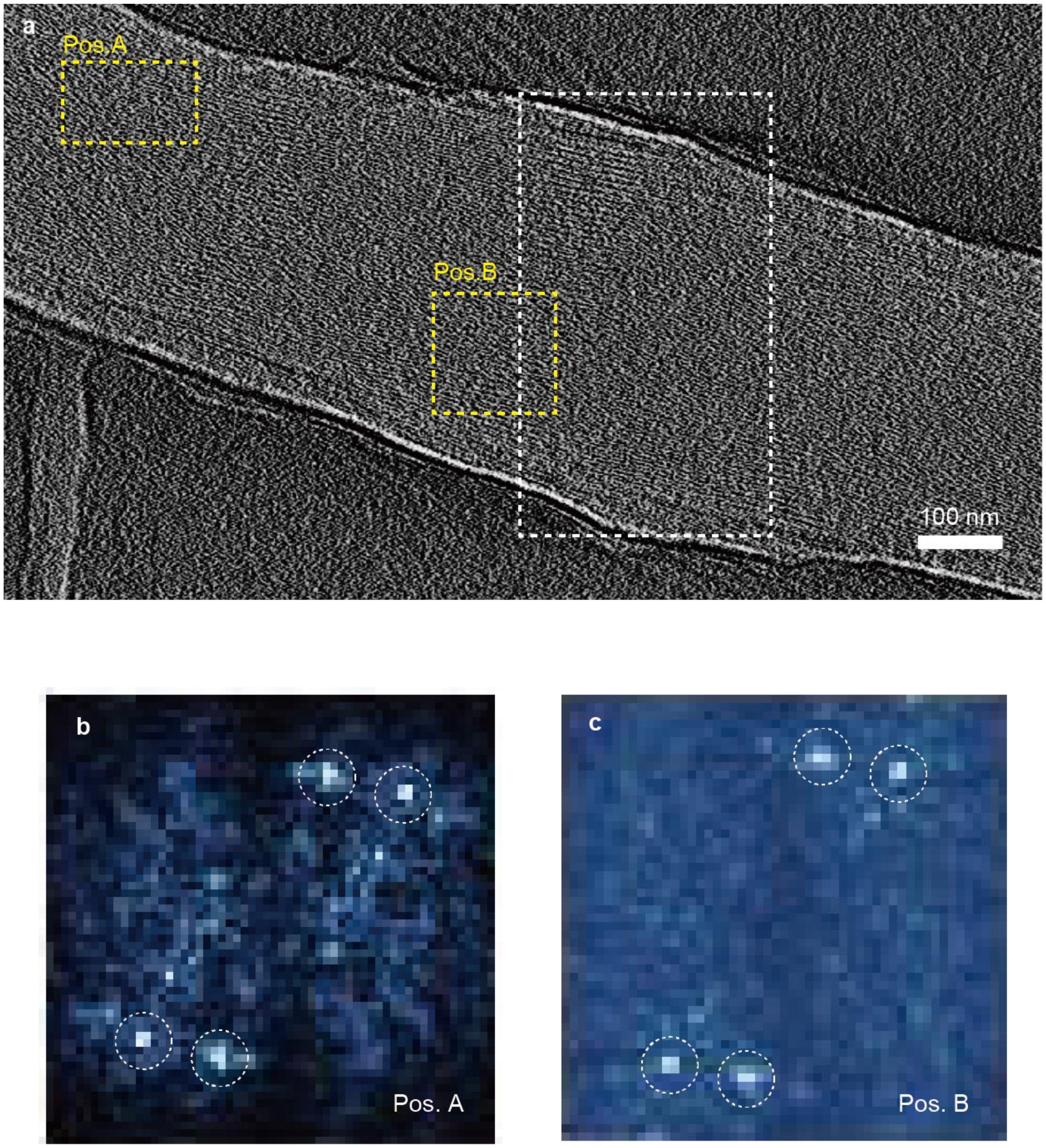

Supplementary Figure 4 │ Additional FFT analysis of the cross-section image of the helical supramolecule. a. A large-scale cross-sectional image of an ET reconstruction of the helical supramolecule. Figure 3a in the main text is an enlarged image represented by white rectangular line. The white scale bar represents 100 nm. **b, c.** FFT patterns of yellow-highlighted areas in (a). As is the case with the analysis in the main text, two pairs of discrete spots are clearly resolved in both FFT results.

Identification of lattice parameters of the helical supramolecules

So far, three different crystal structures of CoPc have been reported (α-, β-, and ε-phases)[1,2]. To reveal a crystal structure of our helical supramolecules, we here perform structural analysis based on rotational electron diffraction patterns of a helical supramolecule. Supplementary Fig. 5a shows a typical TEM image of the helical supramolecule used for the structural analysis. The diffraction pattern obtained from the selected areas highlighted with yellow dashed circles in Supplementary Fig. 5a exhibits a ring-like shape (Supplementary Fig. 5c), which is consistent with the twisting structure described in the main text in which a direction of a crystalline axis changes with different position. The estimated periodicity from the radius of the ring-like shape is 5.06 Å, a slightly larger value than the reported lattice constants along stacking direction (3.75 Å, 4.79 Å, and 4.77 Å for α-, β-, and ε-phases, respectively. See also Table S1.). To further characterize the lattice parameters of our helix, we next focus on the end-terminal branches of unfolded multi-stranded helical supramolecule, where the crystal axes are well-defined (Supplementary Fig. 5b). From the corresponding diffraction pattern (Supplementary Fig. 5d), we can successfully identify the lattice parameters of the supramolecule consisting of helix (Supplementary Table 1). These values are apparently different from the three reported lattice constants of the CoPc crystals, but instead seem to fit a structure of recently found *J*-phase that is fabricated by using PVD technique[3]. A correlation between the crystal phases and an instability towards helical nature is an interesting open issue. The issue is beyond the scope of the present experimental study and we leave this to future independent works.

	α-phase[1]	β-phase[1]	ε-phase[2]	Present work
Space group	P-1	$P2_1/c$	$P2_1/n$	
a (Å)	12.09	14.60	17.84	20.4
b (Å)	3.75	4.79	4.77	4.99
c (Å)	12.80	19.43	15.0	34.9
α (°)	88.96	90	90	90.7
β (°)	90.97	120.78	104.63	90.0
γ (°)	95.09	90	90	90.1

Supplementary Table 1 │ Structural properties of the different phases of CoPc

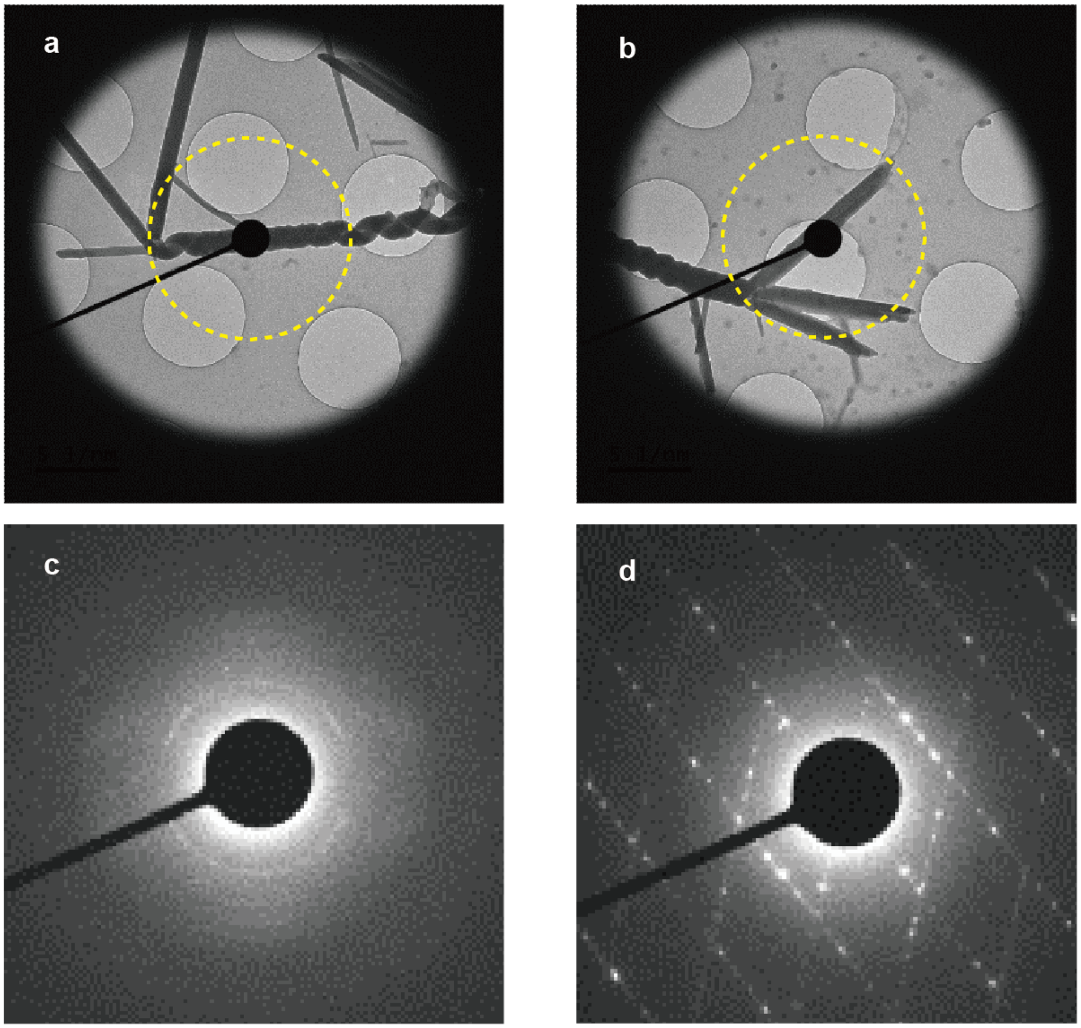

Supplementary Figure 5 │ Structural analysis based on electron diffraction patterns. a, b. Representative TEM images of the target helical supramolecules taken in defocused diffraction mode. Selected areas indicated in yellow dashed circles were used for recording diffraction patterns. **c, d.** Corresponding diffraction patterns obtained from the circled areas in (**a**) and (**b**), respectively. The ring-like intensity at ~ 5 Å resolution in (**c**) is due to the twisting morphology of the supramolecule. From the unfolded branches of the helical supramolecules, the well-defined spot patterns are well resolved as in (**d**). The lattice parameters were determined to a = ~ 5 Å, b = ~ 20.5 Å, c = ~ 35 Å and $\alpha = \beta = \gamma$ = ~ 90° (Supplementary Table 1) from rotational diffraction data sets.

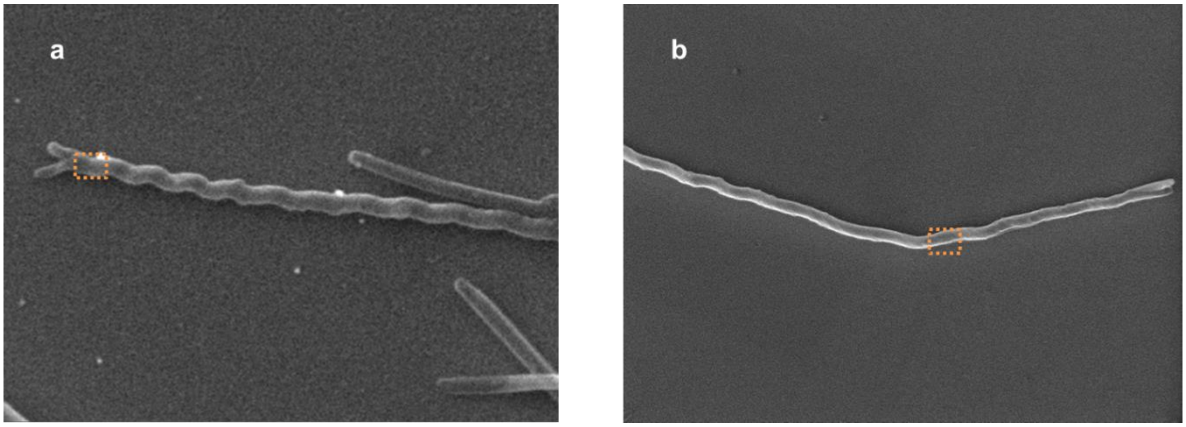

Supplementary Figure 6 │ SEM images of (a) left- and (b) right-handed helical supramolecules used for mC-AFM measurements. *I*-*V* profiles are measured inside the orange-dashed rectangular areas shown in (**a**) and (**b**).

Numerical simulation of 3D fluid dynamics in PVD tube

We performed finite-element simulations (Femtet, Murata Software) to gain insight into 3D fluid dynamics during PVD synthesis, in particular focusing on the possibility that a kind of vortex motion in Ar-gas flow affects on the separation of enantiomer. Parameters used in the simulation are as follows:

- fluid speed : 5×10^{-3} [m/s]
- flow rate : 9.735×10^{-6} [m^3/s]
- dynamic viscosity : 2.360×10^{-5} [m^2/s]

We constructed a geometry of the approximately same size as the setup in our PVD experiment. Supplementary Fig. 7 shows a result of our fluid simulation, in which colored lines represent trajectories of gas flow. Apparently, there is no indication of vortices in flow in whole area. Thus, it is unlikely that a possible vortex-like motion of gas flow leads to a formation of helical structures or their enantioseparation. Our finite-element simulation further supports our claim that CISS-related interaction between helical supramolecules and ferromagnetic substrates is a main origin of the observed enantioseparation.

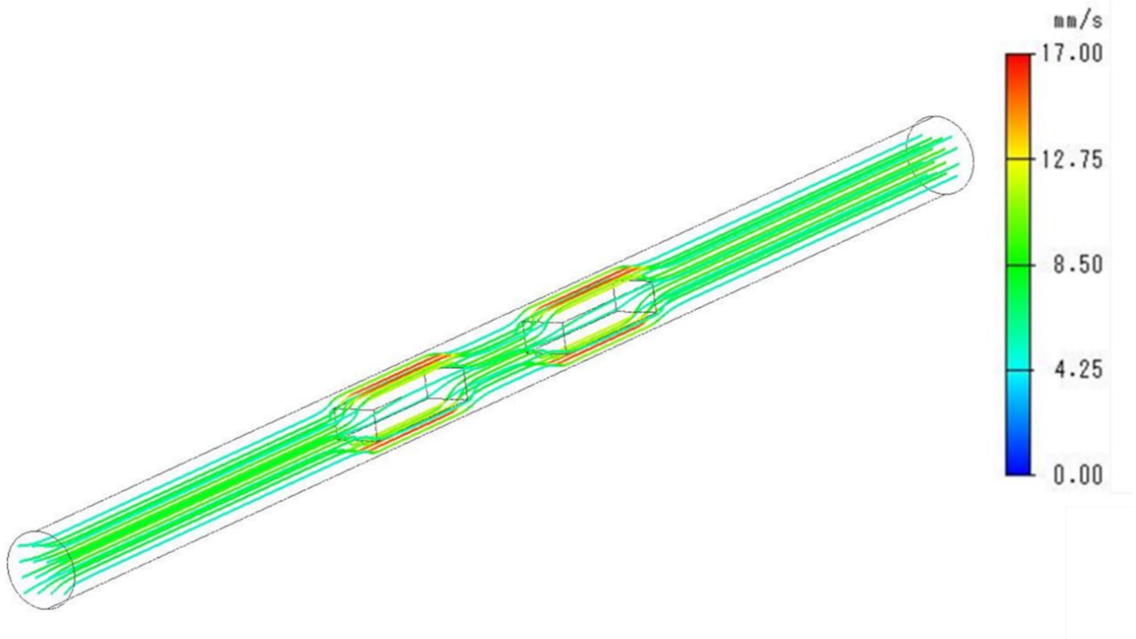

Supplementary Figure 7 │ Results of finite-element fluid simulation.

Supplementary References

1. Ballirano, P., Caminiti, R., Ercolani, C., Maras, A. & Orrù, M. A. X-ray Powder Diffraction Structure Reinvestigation of the α and β Forms of Cobalt Phthalocyanine and Kinetics of the α → β Phase Transition. *J. Am. Chem. Soc.* **120**, 12798–12807 (1998).
2. Linstead, R. P. & Robertson, J. M. The stereochemistry of metallic phthalocyanines. *J. Chem. Soc.* 1736–1738 (1936).
3. Ji, X. *et al.* Cobalt phthalocyanine nanowires: Growth, crystal structure, and optical properties: Cobalt phthalocyanine nanowires: Growth, crystal structure, and optical properties. *Crystal Research and Technology* **51**, 154–159 (2016).
